# X‐ray crystal structures of the type IVb secretion system DotB ATPases

**DOI:** 10.1002/pro.3439

**Published:** 2018-07-18

**Authors:** Marie S. Prevost, Gabriel Waksman

**Affiliations:** ^1^ Institute of Structural and Molecular Biology, University College London and Birkbeck, Malet Street London WC1E 7HX United Kingdom; ^2^ Present address: Channel‐Receptors Unit, Institut Pasteur Paris France

**Keywords:** ATPase, Type IV secretion system, crystal structure, DotB, *Legionella pneumophila*

## Abstract

Human infections by the intracellular bacterial pathogen *Legionella pneumophila* result in a severe form of pneumonia, the Legionnaire's disease. *L. pneumophila* utilizes a Type IVb secretion (T4bS) system termed “*dot/icm*” to secrete protein effectors to the host cytoplasm. The *dot/icm* system is powered at least in part by a functionally critical AAA+ ATPase, a protein called DotB, thought to belong to the VirB11 family of proteins. Here we present the crystal structure of DotB at 3.19 Å resolution, in its hexameric form. We observe that DotB is in fact a structural intermediate between VirB11 and PilT family proteins, with a PAS‐like N‐terminal domain coupled to a RecA‐like C‐terminal domain. It also shares critical structural elements only found in PilT. The structure also reveals two conformers, termed α and β, with an αβαβαβ configuration. The existence of α and β conformers in this class of proteins was confirmed by solving the structure of DotB from another bacterial pathogen, Yersinia, where, intriguingly, we observed an ααβααβ configuration. The two conformers co‐exist regardless of the nucleotide‐bound states of the proteins. Our investigation therefore reveals that these ATPases can adopt a wider range of conformational states than was known before, shedding new light on the extraordinary spectrum of conformations these ATPases can access to carry out their function. Overall, the structure of DotB provides a template for further rational drug design to develop more specific antibiotics to tackle Legionnaire's disease.

PDB Code(s): http://firstglance.jmol.org/fg.htm?mol=Will; http://firstglance.jmol.org/fg.htm?mol=be; http://firstglance.jmol.org/fg.htm?mol=provided

## Introduction

*Legionella pneumophila* is an intracellular bacterial pathogen that is responsible for a severe form of pneumonia in humans termed Legionnaire's disease.^1^ Upon inhalation, the bacteria invade the lung macrophages, and reside in a specialized cytoplasmic vacuole from where they secrete a large set of protein effectors that highjack cellular processes.^2^ Secretion is the result of *Legionella* expressing a specialized Type IVb secretion (T4bS) system, termed the *dot/icm* system, which is apparently related to the bacterial conjugative Type IVa secretion (T4aS) systems.^3^, ^4^ The *dot/icm* system is a double‐membrane spanning channel composed of ∼27 distinct proteins, with a periplasmic/outer membrane core assembly, an inner membrane platform and a cytosolic apparatus.^5^ Homologs of the *dot/icm* system are found in most *Legionella* species, but also in other pathogens, such as *Coxiella burnetii* responsible for Q‐fever,^6^ or *Yersinia pseudotuberculosis* strain IP31758, responsible for the Far East Scarlet‐like fever.^7^

A growing number of studies investigating effectors function in the host cell have revealed that effectors exhibit some degree of redundancy: Indeed, knocking‐out one of them does not necessarily result in a loss of bacterial virulence. Thus, targeting the *Legionella* T4bS system for therapeutic purposes is likely to be more effective than targeting effectors. Indeed, knockouts of genes encoding a few components of the *dot/icm* system are known to produce nonpathogenic strains.^4^ One of them, *dotB*, codes for the main energy supplier of the secretion system, an AAA+ ATPase, homologs to the VirB11 component of the T4aS systems. DotB however appears to be an outlier among VirB11 family proteins. Indeed, while sequence identity between DotB and *A. tumefaciens* VirB11 is high (26%), sequence identity is higher when comparing DotB with the Type IV pilus biogenesis systems ATPase PilT or the Type II secretion system ATPase EpsE (both 31% identity), suggesting that DotB might be more related to PilT/EpsE family proteins than VirB11 family proteins. Type IV pilus biogenesis and Type II secretion employ very similar machineries: It is therefore not surprising that their ATPases should be similar. However, Type IV secretion is thought to be a process very distinct from Type II secretion, involving a completely different sort of apparatus. Thus, its ATPase is believed to be evolutionary unrelated. Were ATPases involved in Types II or IV secretion to be similar, it would suggest evolutionary relationships between these two system types that have not been previously documented.

Here we present the X‐ray crystal structures of two DotB proteins, one from the *dot/icm* system encoded by the *L. pneumophila* genome (termed DotB_L_), at 3.19 Å resolution, and one from the *dot/icm* system encoded in the plasmid of *Y. pseudotuberculosis* IP31758 (termed DotB_Y_), at 2.75 Å resolution. Structurally, all VirB11 protein structures (*Helicobacter pylori* HP0525 or *Brucella suis* VirB11^8^, ^9^, ^10^ solved so far have been very similar. Yet, the two DotB structures presented here reveal a structure much closer to the Type IV pilus biogenesis ATPases than to VirB11. These structures thus provide new insights on the evolutionary relationship between secretion systems and also clues to the understanding of the mechanism of ATP‐driven conformational changes leading to the energization of the system. These structures will also prove useful in future drug‐design efforts aiming to efficiently target *Legionella* infections.

## Results and Discussion

### The overall structure of DotB reveals a close relationship with the PilT ATPases

DotB_L_ and DotB_Y_ overexpressed well in *E. coli*, and they both crystallized readily overnight in various conditions, generating crystals diffracting to circa 8 Å resolution. Conditions optimization, including the addition of AMP–PNP for DotB_L_, yielded crystals diffracting to 3.19 and 2.75 Å resolution, respectively (Table 1). Both crystals belonged to the P1 space group, with 12 subunits in the asymmetric unit for DotB_L_ and 6 for DotB_Y_. The two structures were solved by molecular replacement using a search model combining structures of PilT and PilT2 (see Materials and Methods). For the DotB_L_ structure, two identical hexamers could be built, on top of each other, while only one is present in DotB_Y_. For DotB_L_, the 12 chains were built from residue 5 ± 1 to 373 ± 1 depending on the chain with some interruption in the β4–β5 loop, while for DotB_Y_, the 6 chains are resolved from residue 3 ± 2 to 388, covering in both cases more than 99% of the protein sequence.

**Table 1:** Data Collection and Refinement Statistics

	DotB_L_	DotB_Y_
Wavelength	0.97625	0.97626
Resolution range	36.04–3.19 (3.31–3.19)	40.57–2.75 (2.85–2.75)
Space group	P 1	P 1
Unit cell (*a*, *b*, *c*, *α*, *β*, *γ*)	109.2, 109.3, 119.8, 83.7, 86.6, 60.7	83.0, 93.6, 109.9, 103.9, 102.0, 99.9
Total reflections	273,289 (25,672)	269,969 (24,532)
Unique reflections	78,104 (7,560)	77,292 (7,305)
Multiplicity	3.5 (3.4)	3.5 (3.4)
Completeness (%)	98.0 (95.3)	97.8 (91.5)
Mean *I*/sigma (*I*)	6.95 (0.84)	10.33 (1.61)
Wilson B‐factor (Å^2^)	102.40	69.97
*R*‐meas	0.2246 (3.094)	0.1008 (0.8582)
CC1/2	0.99 (0.245)	0.996 (0.656)
Reflections used in refinement	78,095 (7,560)	77,292 (7,305)
*R*‐work	0.2333	0.2546
*R*‐free	0.2613	0.2961
CC (work)	0.930 (0.384)	0.937 (0.601)
CC (free)	0.925 (0.342)	0.948 (0.624)
Number of nonhydrogen atoms	34,670	18,021
Macromolecules	34,610	18,021
Ligands	60	0
Protein residues	4,420	2,313
Rmsd (bonds, Å)	0.016	0.013
Rmsd (angles, deg)	1.53	1.13
Ramachandran favored (%)	95	94
Ramachandran allowed (%)	4.4	5
Ramachandran outliers (%)	0.6	1
Rotamer outliers (%)	6.2	12
Clashscore	41.13	53.71
Average B‐factor (Å^2^)	67.74	44.31
Macromolecules (Å^2^)	67.71	44.31
Ligands (Å^2^)	83.87	–
Number of TLS groups	12	6

Statistics for the highest‐resolution shell are shown in parentheses.

**Figure 1:**
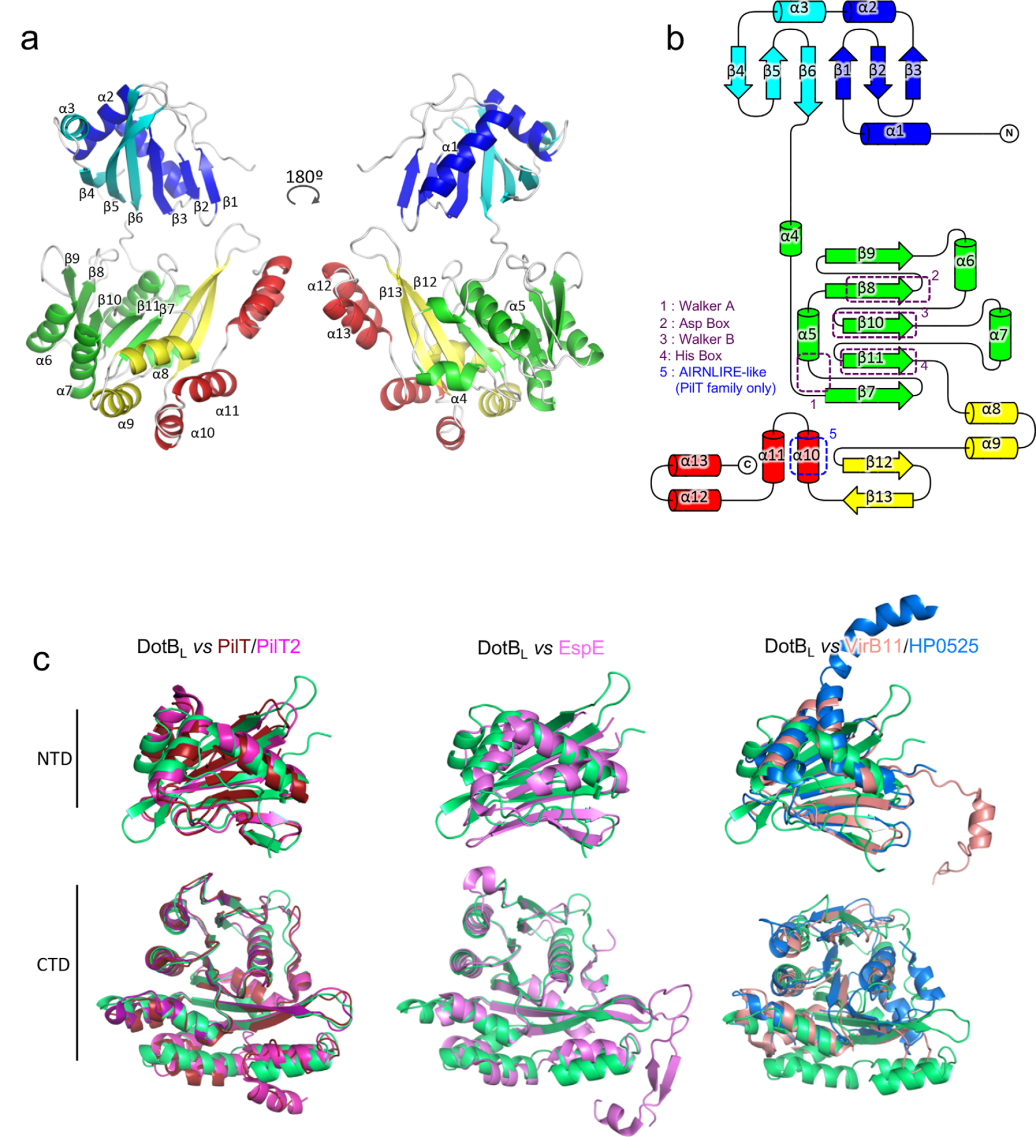
DotB_L_ crystal structure. (A) DotB_L_ subunit structure. A single subunit in the α‐conformation is depicted in cartoon representation in two views rotated by 180°. Secondary structure elements are labeled and colored according to sub‐domains of the monomer. (B) Topology diagram of a DotB_L_ subunit. β‐strands are represented by arrows, α‐helices by cylinders. The elements are colored as in a. Conserved regions are highlighted by dashed boxes. (C) Fold comparison of the NTD and CTD domains of DotB_L_ with other secretion ATPases. Domains were aligned using the Cα of their conserved elements. From left to right: DotB_L_ (green) aligned with PilT (red, pdb 2EWV) and PilT2 (magenta, pdb 5FL3), with EspE (pink, pdb 1P9R) and with *B. suis* VirB11 (light pink, pdb 2GZA) and HP0525 (blue, pdb 1NLY). The N‐terminal domain of DotBL aligns with that of Pilt2, PilT, HP0525, *B. suis* VirB11 and EspE with an rmsd in Cα atoms of 1.4, 2.0, 4.1, 4.9, and 1.4 Å, respectively while the C‐terminal domain aligns with that of Pilt2, PilT, HP0525, *B. suis* VirB11 and EspE with an rmsd in Cα atoms of 0.8, 0.8, 5.3, 3.3, and 1.8, respectively.

DotB_L_ and DotB_Y_ subunits display a very similar topology. A DotB_L or Y_ subunit is composed of a ∼150 aminoacids long N‐terminal domain (NTD), comprising 6 β‐strands and 3 α‐helices, and a ∼250 aminoacids C‐terminal domain (CTD) consisting of 7 β‐strands and 10 α‐helices [Fig. 1(A,B)]. The two domains are connected by a 10 aminoacids proline‐rich linker. The solvent‐oriented loop between β4 and β5 is variable within the hexamer, probably due to local differences in crystal contacts. DotB_L_ and DotB_Y_ monomer structures align very well with a root mean square deviation (rmsd) in Cα atoms of 0.9 Å [Supporting Information Figs. S1(A) and S2].

A DotB_L_ subunit readily aligns with a PilT2 subunit from *T. thermophilus* (PDB code 5FL3) with a rmsd in Cα atoms of 0.77 Å [Supporting Information Figs. S1(B), S1(C), and S2], with mainly differences in domain orientations. The same alignment using the *A. aeolicus* PilT, the *V. cholerae* EpsE and the *H. pylori* VirB11 homologue HP0525 results in rmsd of 0.8, 1.8, and 3.4 Å, respectively, suggesting that DotB family proteins are structurally more similar to PilT or EpsE family proteins, than VirB11 proteins [Supporting Information Figs. S1(C–E) and S2]. Given the similarity in structure between DotB_L_ and DotB_Y_, we will focus the description of the structure on DotB_L_, highlighting differences with DotB_Y_ only when necessary.

The NTD of DotB_L_ adopts a PAS‐like fold, similar to PilT, EpsE, and HP0525 [Fig. 1(C)]. The main part of the CTD adopts a RecA fold and harbours the signature motifs of the AAA+ family: The Walker A motif between β7 and α5 (also referred as P‐loop), the Asp Box on β8, the Walker B motif on β10 and the His Box on β11. The rest of the CTD consists of 4 α‐helices, absent in VirB11 homologs, and less well conserved in PilT and EpsE. The first of those 4 helices, α10, harbors a motif similar to the one observed in PilT, namely the AIRNLIRE motif (EVRDILLE in DotB), and previously described as a PilT signature required for pilus retraction [Fig. 1(B,C); Supporting Information S2].^11^

### DotB_L_ forms an asymmetric hexamer

**Figure 2:**
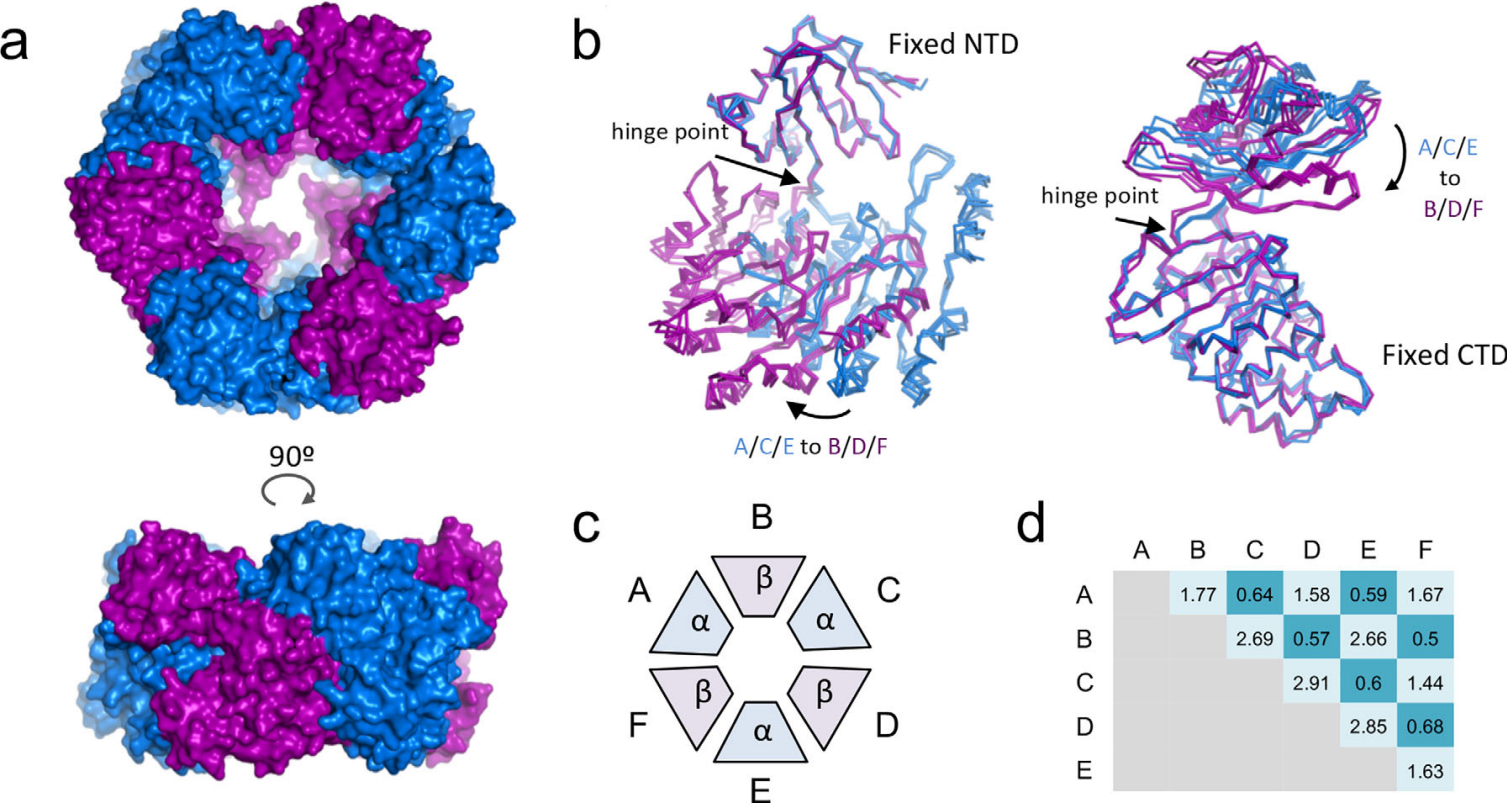
The DotB_L_ hexamer is formed of trimers of α and β dimers. (A) Top (top subpanel) and side (bottom subpanel) views of the DotB_L_ hexamer in surface representation. Subunits A, C, and E, which are in the α conformation, are colored in blue, while subunits B, D, and F, which are in the β conformation, are colored in purple. (B) Superposition of the α and β conformers. Subunits are shown in ribbon representation, color‐coded as in a, that is, α in blue and β in purple. The superposition was obtained by aligning the NTDs (left) or the CTDs (right). The straight and curve arrows identify the hinge point and the direction of the α to β transition, respectively. (C) Schematic representation of the subunits organization within the hexamer. (D) Summary of rmsd values (Å) resulting from the alignment of the two designated subunits.

While all the subunits in the structures adopt the same fold, the subunits within the hexameric assembly display differences in domains orientation [Fig. 2(A)]. When aligning the subunits by their NTDs, we observed two orientations for the CTD, one (which we termed “α”) where the linker is aligned with the central β‐strand of the NTD β6, and the other (which we termed “β”) where the whole CTD is rotated by 46° towards the *n*‐1 subunit and the center of the hexamer [Fig. 2(B)]. The hinge point of this rotation is within the proline‐rich linker. In the hexamer, subunits A, C, and E adopt the α conformation, and subunits B, D, and F the β conformation, yielding an overall threefold symmetry [Fig. 2(C)]. Rmsd calculated from the alignment of the subunits by their Cα atoms illustrate that pattern, with subunits in the same conformation having an rmsd ≤0.7 Å [Fig. 2(D)]. In DotB_Y_, A, B, D, and E are in the α conformation, while C and F are in the β one, yielding an overall twofold symmetry (Supporting Information Fig. S3).

To understand the implications of this conformational change over the entire hexamer, we generated a model in which each subunit *n* is made to adopt the conformation of the *n +* 1 adjacent subunit (Supporting Information Movie S1). Looking at the resulting transition within the hexamer, it becomes apparent that the interface between the NTD of subunit *n* and the CTD of subunit *n +* 1 is remarkably unaffected by the conformational change: Rotation around the 6 NTD_*n*_/CTD_*n+*__1_ interfaces within the hexamer leads the CTDs to slide in or away from the center of the hexamer. The same procedure applied to the structure of DotB_Y_ leads to similar observations with the NTD_*n*_/CTD_*n+*__1_ interface between two subunits remaining unaffected while the CTDs move in and out as a result (Supporting Information Movie S2). Structural investigations of Type II secretion ATPases and Type IV pilus biogenesis motors reported similar observations.^12^, ^13^, ^14^

### Protein–protein interactions within the hexamer

**Figure 3:**
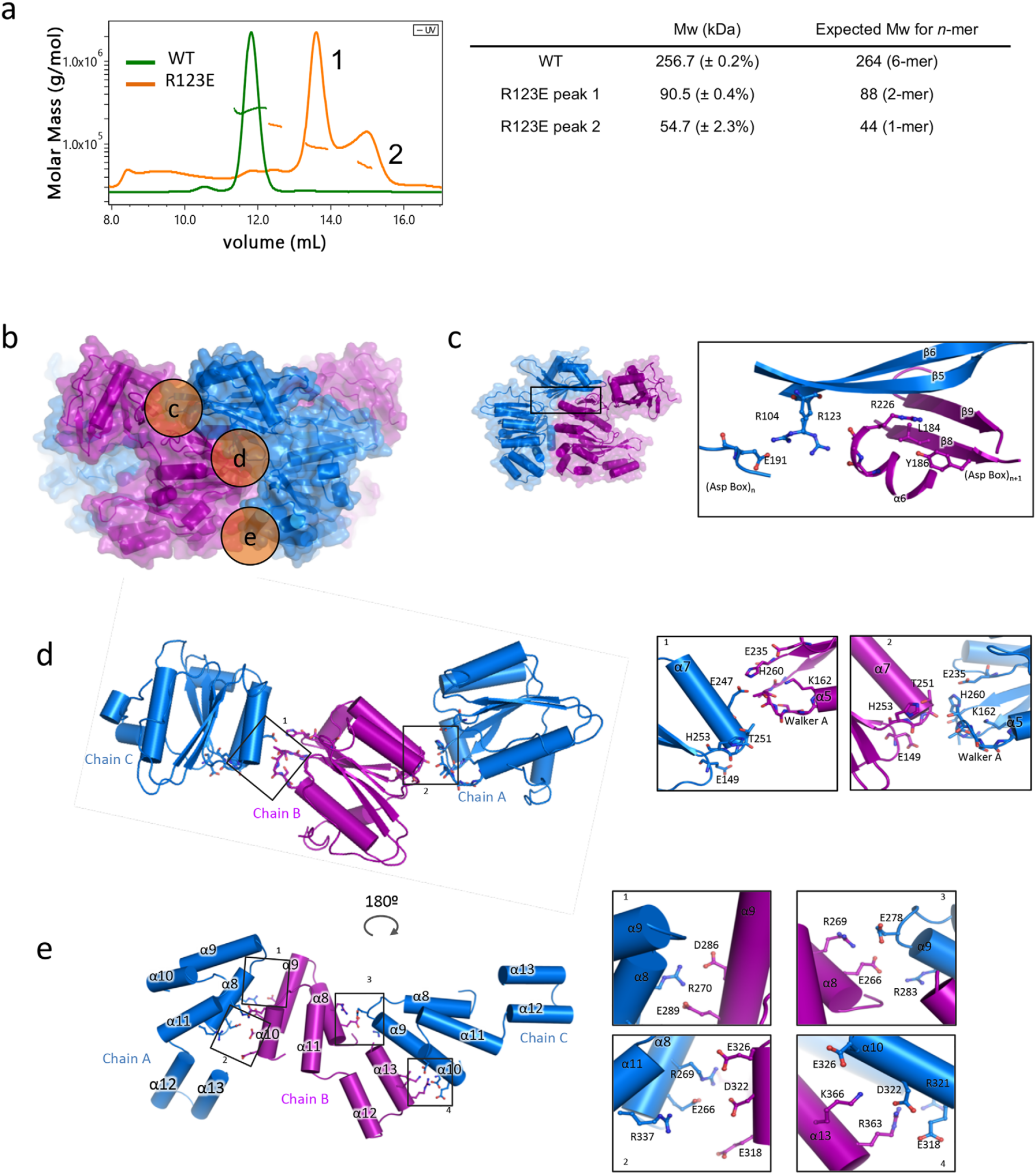
Protein–protein interfaces between subunits. (A) SEC–MALS profiles of the purified DotB_L_ wild‐type (WT in green) and the R123E DotB_L_ mutant (in orange), and the corresponding calculated and expected masses. (B) Locations of three of the DotB_L_ intersubunit interfaces detailed in panels c, d, and e. Side views of two subunits in the α (blue) and β (purple) conformations are shown in stronger colors. The location of the regions described in panels c, d, and e is shown in orange circles labeled correspondingly. (C) NTD_*n*_/CTD_*n*__+1_ interface (region c in panel B). Left: Location of the interface shown at right. The α–β dimer is shown as in B. The box locates the region detailed in the zoom‐in view at right. Right: Close‐up view of the interface. Secondary structure elements and side chains involved in intersubunit contacts are shown in cartoon and stick representation, respectively, and labelled. (D) Upper part of the CTD_*n*_/CTD_*n*__+1_ interface (region d in panel B). Left: Overview of the interface. CTDs of three subunits are in cartoon representation. Side chains involved in intersubunit interactions are shown in stick representation. Boxes indicate the zoom‐in regions shown at right. Right: Close‐up view of the regions shown in boxes. Representation and labeling are as in C. (E) Lower part of the CTD_*n*_/CTD_*n*__+1_ interface (region e in panel B). Left: Overview of the interface. Representation and labeling are as in D. Right: Close‐up view of the regions shown in boxes. Representation and labeling are as in D.

To assess the oligomeric state of DotB_L_, we performed SEC–MALS and calculated a mass of 256.7 ± 0.2% kDa [Fig. 3(A)], which corresponds to a hexameric DotB_L_ (the monomer's MW is 44 kDa). Thus, DotB_L_ was expressed and crystallized in its biological oligomeric state. In this section, we describe further the various interfaces that hold the hexamer together.

In the structure, within the hexamer, the 6 NTDs and the 6 CTDs form two superposed rings [Fig. 3(B)]. Because of the two alternating conformations [α and β (see above)] the subunits can adopt along the hexamer, the contacts areas can be subdivided between those between α and β subunits or between α–β dimers. The NTD_*n*_/NTD_*n+*__1_ within the α–β dimer is small (127 Å^2^, Table 2), but, remarkably, there are no contacts between adjacent NTDs between two α–β dimers (Table 2). Overall, the NTDs are rather poorly stabilized by NTD/NTD interactions as observed in previous VirB11 homolog structures.^8^, ^9^, ^10^ Contacts between CTDs within or between α–β dimers are also different with contact surface areas between two α–β dimers almost double the size of those within the α–β dimer (Table 2). Finally, although contacts between NTDs and CTDs within subunits are substantial and larger in the β conformation than in the α conformation, contacts surface areas are much larger between NTDs and CTDs of adjacent subunits (NTD_*n*_/CTD_*n*__+1_; Table 2).

**Table 2:** Interface Surface Areas Between Domains: Surfaces are Calculated by the PDBePISA Server^30^

DotB_L_ Interface	Surface (Å^2^)	Number of residues involved	
NTD_*n*_–NTD_*n+*__1_ interfaces			
NTD_A_–NTD_B_	127.0		9
NTD_B_–NTD_C_	0		0
CTD_*n*_–CTD_*n*__+1_ interfaces			
CTD_A_–CTD_B_	386.5		35
CTD_B_–CTD_C_	617.7		39
NTD_*n*_–CTD_*n*_ interfaces			
NTD_A_–CTD_A_	343.3		20
NTD_B_–CTD_B_	515.6		26
NTD_C_–CTD_C_	328.5		17
NTD_*n*_–CTD_*n+*__1_ interfaces			
NTD_A_–CTD_B_	1102.7		61
NTD_B_–CTD_C_	1012.6		57

DotB_Y_ Interface	Surface (Å^2^)	Number of residues involved	
NTD_*n*_–NTD_*n*__+1_ interfaces			
NTD_A_–NTD_B_	20.6		3
NTD_B_–NTD_C_	124.1		8
NTD_C_–NTD_D_	51.5		3
CTD_*n*_–CTD_*n*__+1_ interfaces			
CTD_A_–CTD_B_	594.0		42
CTD_B_–CTD_C_	548.4		43
CTD_C_–CTD_D_	629.4		36
NTD_*n*_–CTD_*n*_ interfaces			
NTD_A_–CTD_A_	352.5		24
NTD_B_–CTD_B_	347.2		25
NTD_C_–CTD_C_	516.0		30
NTD_D_–CTD_D_	356.0		23
NTD_*n*_–CTD_*n*__+1_ interfaces			
NTD_A_–CTD_B_	1119.4		63
NTD_B_–CTD_C_	1094.5		64
NTD_C_–CTD_D_	1159.0		65

### NTD_*n*_/CTD_*n*__+1_ interactions

This interface is large and conserved all along the hexameric assembly [Fig. 3(C)]. Main contacts are between residues in the β5 and β6 strands of NTD_*n*_ and residues in β8 (Asp Box), β9 and α6 (before Walker B) of CTD_*n+*__1_. NTD residues involved in the interface are polar or charged, with three residues well conserved in the family (Arg104, Asn106 and Arg123). In the CTD, the residues are also well conserved and close or within motifs involved in ATP hydrolysis. As mentioned above, this interface remains unaffected by the α to β transition.

To assess how important these interactions might be, we performed single mutations at the interface. We chose to target Arg104 and Arg123 because their side chains lie at the center of the NTD_*n*_/CTD_*n*_/CTD_*n*__+1_ interface [Fig. 3(C)]. Arg123 interacts with carboxyl groups of residues at the C‐terminal end of α6 in CTD_*n*__+1_, while Arg104 side chain interacts with Glu191 in CTD_*n*_. We mutated the two arginines into glutamates and expressed the resulting mutant proteins in *E. coli*. At the gel filtration step, the two mutants elute at a later volume than the wild‐type protein, in two peaks with variable ratios depending on the batch. We performed SEC–MALS on the R123E mutant and identified those two peaks as being dimeric and monomeric forms of DotB_L_ [Fig. 3(A)]. We also observed that the two mutants precipitate quickly after purification at 4°C while the wild‐type protein is stable for several days. Overall, we concluded that disruption of this interface by mutating those two arginines leads to disruption of the hexamer. Interestingly, dimers are observed, confirming the structural observation that interfaces within or between α–β dimers are different.

### The CTD_*n*_/CTD_*n*__+1_ interface

In the CTD_*n*_/CTD_*n*__+1_ interface, the closest contacts are made in two regions [d and e in Fig. 3(B)]: One in the upper part (nearest the NTDs) consisting of residues in the Walker A region of CTD_*n*_ and in α7 of CTD_*n*__+1_ *; and the* other in the lower part (furthest from the NTDs) consisting of residues in various helices on each side (see details below). For both these regions, the intersubunit interactions networks are different within or between α–β dimers, thus defining two CTD_*n*_/CTD_*n*__+1_ interfaces.

In region d, in the interface between α–β dimers [i.e., between chains B and C in Fig. 3(D)], the Walker A motif region of CTD_*n*_ contacts α7 of CTD_*n*__+1_ in its middle part. In contrast, in that same region but in the interface within α–β dimers [i.e., between chains A and B in Fig. 3(D)], the Walker A motif makes contact with the C terminal part of that same helix. The Walker A motif conserved polar residues together with Glu235 (Walker B) and His260 (His box) are indeed in close proximity with Glu247 and Thr251 in the interface between α–β dimers, but contact Thr251, His253, and Glu149 (β7) in the interface within α–β dimers.

In region e, the CTD_*n*_/CTD_*n*__+1_ interfaces between or within α–β dimers are very different. Indeed, in subunits adopting a β conformation, the helical bundle consisting of α8, 9, 10, and 11 is pushed towards the center of the hexamer compared to the same region in the α conformation subunits. This profoundly remodels interactions between α8/11/13 on one side and α9/10 on the other [Fig. 3(E)]. More specifically, at the CTD_*n*_/CTD_*n*__+1_ interface within α–β dimers [i.e., chains A and B in Fig. 3(E)], intersubunit contacts are made by side chains from α8 and α11 (in chain A which is in the α conformation) and from α9 and α10 (in chain B which is in the β conformation). We observed two clusters of charged residues, Arg270 (in chain A) facing Asp286 and Glu289 (in chain B), and Arg269, Glu266 and Arg337 (in chain A) facing Glu326, Asp322, and Glu318 (in chain B). No residues in helix α13 is involved in contact. This is in contrast with the CTD_*n*_/CTD_*n*__+1_ interface between α–β dimers [i.e., chains B and C in Fig. 3(E)], where residues in α13 play important roles: Indeed, a cluster of interactions is observed between Lys366 and Arg363 of α13 in chain B (in β conformation) and Glu326, Asp322, Arg321, and Glu318 in α10 of chain C (in α conformation). Another interaction cluster involves Arg269 and Glu266 in α8 of chain B and Glu278 and Arg283 of α9 in chain C. Overall, helices α8 and α10 appear most involved in both types of CTD_*n*_/CTD_*n*__+1_ interfaces. Interestingly, from the 6 last α‐helices of the CTD, α8, and α10 are the best conserved within the family; α8 follow the His box and α10 holds the AIRNLIRE‐like motif, required for proper function in PilT ATPases. In PilT, mutations on the AIRNLIRE motif do not disrupt hexameric oligomerization *in vitro*,^11^ but do induce a loss of function *in vivo*.

As mentioned above, the DotB_Y_ hexamer differs from the DotB_L_ hexamer in exhibiting an ααβααβ arrangement instead of the αβαβαβ arrangement observed in DotB_L_. A superposition of the DotB_Y_ α–β and α–α dimers reveals that, in the DotB_Y_ structure, the CTD_*n*_/CTD_*n+*__1_ interactions are essentially the same in both dimers [Supporting Information Figs. S3(E) and S3(F)]. However, the NTD_*n*_/NTD_*n*__+1_ interactions are different with only very few contacts observed between NTDs in the α–α dimers.

### Active site of DotB

**Figure 4:**
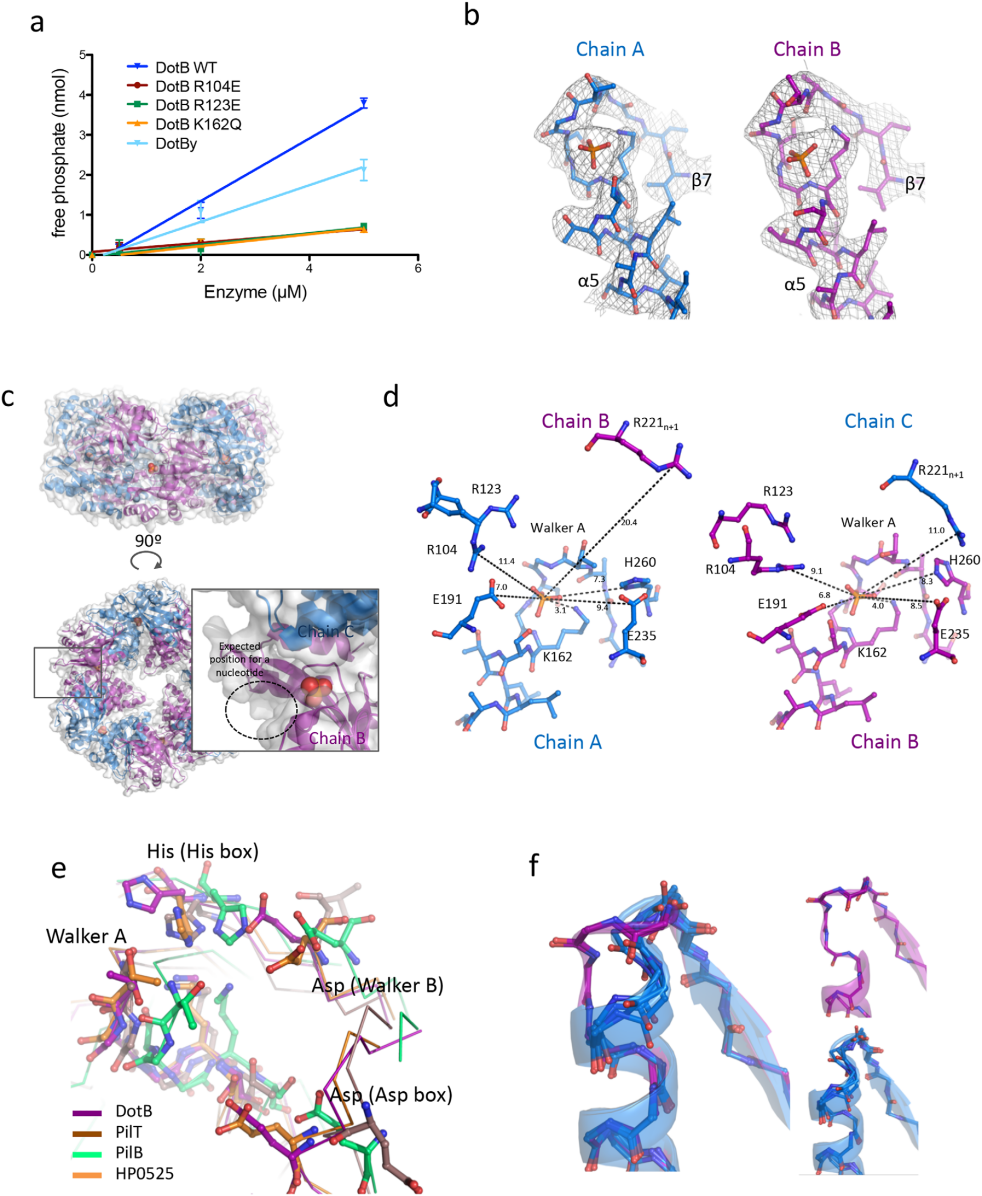
The DotB_L_ active site. (A) ATPase activity assays. Briefly, various concentrations of DotB_L_ and DotB_Y_ wild‐type and mutant proteins were incubated with ATP for 1, 2, and 5 min, and the resulting free phosphate concentrations were determined. Errors bars indicate the standard deviations from three experiments. (B) Electron density at the active site. The map (in gray chicken wire) was calculated using 2Fo–Fc coefficients and phases derived from the finally refined model) and contoured at 1.5σ level. The region shown is between residue 154 and residue 170. The model is shown in stick representation with atoms color‐coded red for oxygen, light blue for carbon, dark blue for nitrogen, and orange for phosphorus. (C) Location of the phosphate within the DotB_L_ hexamer. The hexamer is in cartoon and semi‐transparent surface representations with the phosphate groups shown as spheres. Side (upper sub‐panel) and top (lower left panel) views are shown. The box in the lower left panel locates the region which is shown in more details in the zoom‐up window to the right. In this window, the NTDs have been removed to gain an unobstructed view of the ATP‐binding site. A dashed circle indicates the expected position of a nucleotide. (D) Residues involved in the active site in the α‐subunit (left) and the neighboring β‐subunit (right). Indicated distances are in Å. Side and main chains are in stick representation. (E) Comparison of the DotB_L_ active site with PilT (pdb 2EWV), PilB (pdb 5IT5), and HP0525 (pdb 2GZA). Conserved residues are shown in ball‐and‐stick representation. (F) The DotB_Y_ Walker A region. The main chains of the Walker A regions of the six subunits are shown shown in ball‐and‐stick and semitransparent cartoon representations. Chains A, B, D, and E are in blue (α conformation), chains C and F are in purple (β conformation). α and β conformations are shown separately on the right.

To make sure that the crystallized proteins are functional, we assayed their ability to hydrolyze ATP [Fig. 4(A)]. Both purified DotB_L_ and DotB_Y_ hydrolyze ATP, demonstrating that the purification procedure did not affect their biological activity. Kinetic measurements on DotB_L_ yielded a Km of 0.99 ± 0.30 m*M* and a *k*_cat_ of 674 ± 50 s^−1^. In the early stages of refinement, a positive density in the Fo‐Fc omit map repeatedly emerged at the CTD_*n*_/NTD_*n*_ interface, at the N‐terminal end of α5 where the Walker A motif or P‐loop is [Fig. 4(B,C)]. In the final stages, we could model at this position a phosphate group in all chains [Fig. 4(B)]. DotB_L_ was crystallized in the presence of AMP–PNP and ATPase activity assays using DotB_L_ preincubated with increasing concentrations of AMP‐PNP show that the analogue binds and competes with ATP resulting in an inhibition of phosphate release (Supporting Information Fig. S4). Yet, we could observe density for only phosphate not AMP–PNP.

In the β‐subunits, the phosphate group binds to the N‐terminal end of α5 [Fig. 4(D)]. The phosphate is clamped by the main chain NH groups between residues 158 and 163 (TGSGKS) and by the Lys162 side chain. The importance of Lys162 in ATP‐binding and hydrolysis was confirmed by assaying a single mutant K162Q for its ability to hydrolyze ATP [Fig. 4(A)]. In the vicinity of this binding site (less than 6 Å away), the side chains of Glu191 (Asp box), Glu235 (Walker B) and His260 (His box), together with Arg221 (in α6) from the next subunit, point towards the ligand. In the α‐subunits, the binding site is highly similar, except for *n +* 1 Arg221, which is further from the binding site due to the alternate conformation of the *n +* 1 subunit [Fig. 4(D)].

Superposition of the ATP‐binding site of DotB_L_ with structures of liganded PilT, PilB and VirB11 suggests a well‐conserved binding mode among these ATPases [Fig. 4(E)], the phosphate group in DotB_L_ binding where the β phosphate of a nucleotide would bind. Conserved features include: The P‐loop main and side chains, the conserved histidine residue form the His Box, and aspartate residues from the Walker B and the Asp Box. Structures of PilT and the VirB11 homolog HP0525 in various nucleotide‐bound or unbound states suggest the involvement of homologous residues in DotB_L_, namely Arg104 (in β5) and Arg123 (in β6) from the NTD, in ligand binding:^9^, ^15^ in the ATP‐bound conformation, these arginines coordinate the gamma phosphate of the nucleotide, while in the Apo or ADP‐bound conformations (where the NTD and the CTD within each subunit are further apart), those residues are more than 8Å away like they are in DotB_L_. This suggests that DotB_L_ will likely cycle through similar conformational changes affecting the NTD/CTD relative orientations during ATP‐binding, ‐hydrolysis and ‐release. Consistent with this model is the observation that two DotB_L_ mutants, R104E and R123E, are inactive [Fig. 4(A)]. However, whether this is caused by defects in oligomerization (see above) or impairment of the active site remains to be clarified.

The DotB_Y_ protein was crystallized without any phosphate or nucleotide in the buffer. Nevertheless, when analyzing the ATP‐binding site region of all six subunits, we observed a striking difference between chains adopting the α and β conformations [Fig. 4(F)]. In the β conformation (chains C and F), the Walker A region is similar to what is observed in the bound structures of DotB_L_ and other ATPases, namely we observe an unwinding of the first α‐helical turn of α5 rich in Ser/Gly that provides space for a phosphate to bind. Surprisingly, in the α conformation of DotBy (chains A, B, D, and E), this α‐helical turn is present, formed with the carboxyl groups of residues Ser173 and Ser174 (equivalent to Ser159 and Gly160 in DotB_L_) contacting the NH groups of Ser177 and Thr178 respectively. With such configuration, a phosphate group would not have the space to bind at this position. Interestingly, in the structures of PilT, PilT2, PilB, EpsE, and VirB11, this additional α‐helical turn of the P‐loop is not observed.

### Mapping mutations on the DotB_L_ structure explains their phenotype

**Figure 5:**
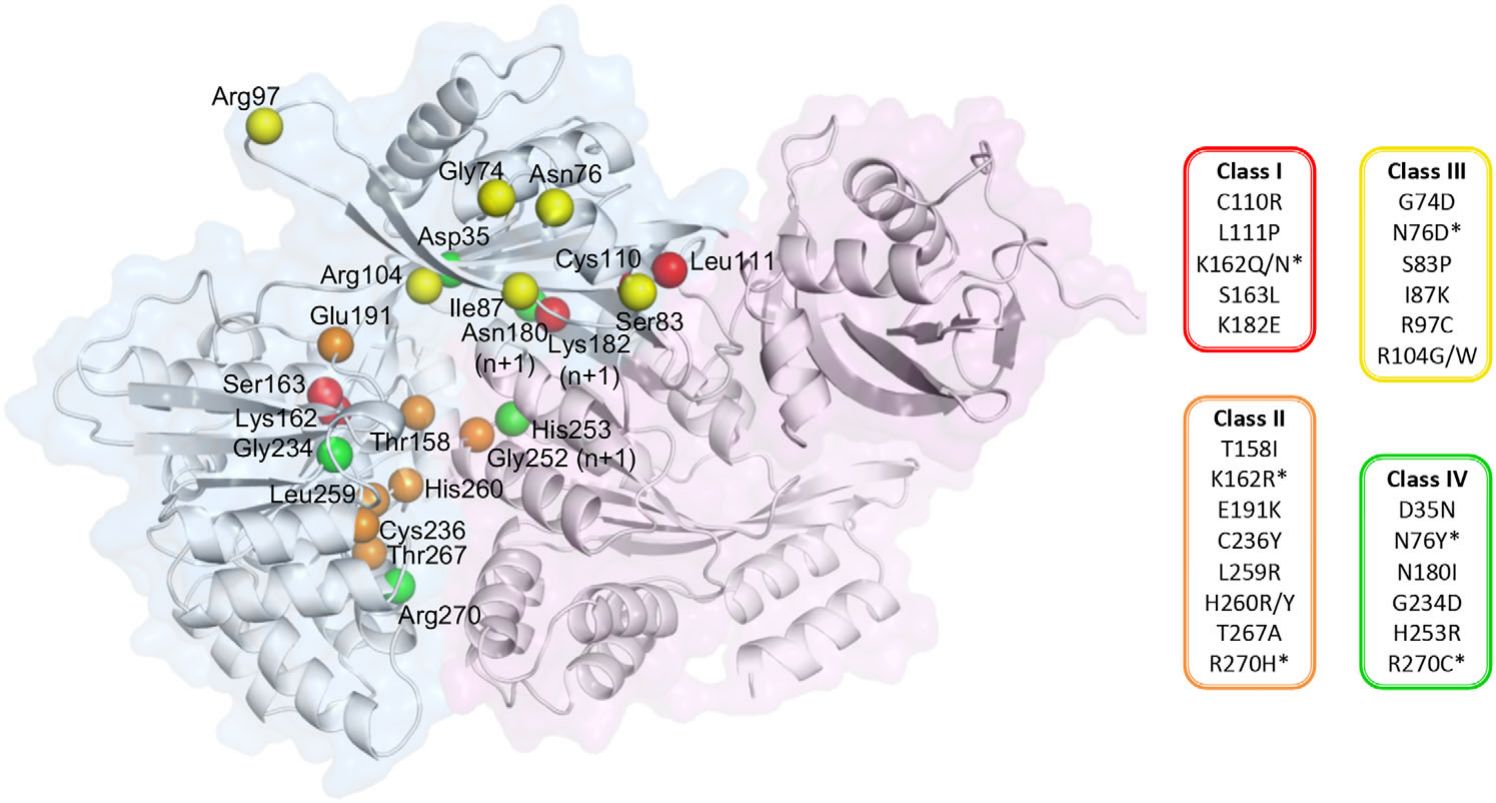
Mapping of DotB mutants. Two adjacent subunits are depicted in cartoon and surface representations, with an α‐subunit in blue and the adjacent β‐subunit in purple. The locations of residues mutated in *L. pneumophila* DotB by Sexton *et al*. (2005) are identified by their Cα shown as spheres, color‐coded according to the phenotype classes listed on the right (see main text). The * indicates that the mutated residues are present in more than one class depending on the nature of the substitution.

In 2005, Sexton *et al*.^16^ published a genetic screen of single mutations of DotB, testing the ability of a Δ*dotb L. pneumophila* strain complemented by the mutants to: (1) grow inside U397 cells, (2) bind ATP (using ATP agarose beads) and (3) hexamerize (as assessed by native PAGE). They described four classes of mutants defined as “Class I: Mutants with a known biochemical defect; Class II: Mutants with a predicted enzymatic defect; Class III: Mutants with an unknown defect; and Class IV: Mutants with partial functionality”. We examined the position of these mutated residues in the DotB_L_ structure (Fig. 5).

Mapping class II mutants on the DotB_L_ structure shows that all residues are indeed in the vicinity of the active site. To those mutants, we can add K162N/Q and S163L (Class I) and G234D and H253R (Class IV) also in close proximity to the active site.

Three Class I and two Class IV mutants (C110R, L111P, K182E, and D35N, N180I, respectively) are reported to have hexamerization defects, and are indeed located at the NTD_*n*_/CTD_*n*__+1_ interface, as are the R104E and R123E mutants of the present study.

Finally, most mutants from Class III are located at the NTD_*n*_/CTD_*n*__+1_ interface but are still able to bind ATP and to hexamerize. We propose that those mutants could affect the ability of DotB subunits to switch from the α to the β conformation, since they are located in the interface that remains unaffected by the α to β transition (Fig. 5; Supporting Information Movie S1). Similarly, the mutant R270C, partially functional, could harbor a loss of function due a conformation‐switch defect, since Arg270 is part of the critical lower CTD_*n*_/CTD_*n*__+1_ interface.

## Conclusion

The dot/icm T4bS system of *L. pneumophila* is powered by two ATPases: The membrane protein DotL, which is part of the coupling sub‐complex responsible for the recruitment of substrates and their delivery to the secretion channel^17^ and DotB, potentially involved in complex assembly and secretion. DotB is related in sequence to the VirB11 ATPases of conjugative T4aS systems.

In this study, we solved the X‐ray structures of two DotB homologues, DotB_L_ and DotB_Y_. They both exhibit a classical AAA+ ATPase fold, with a PAS‐like N‐terminal domain and a RecA‐like ATPase domain. In structure, they are however more related to the PilT family of proteins involved in Type IV pilus biogenesis and EspE family of proteins involved Type II secretion. In both DotB structures, the proteins can assume two distinct conformations, α and β, that differ in their NTD/CTD orientations. We hypothesize that DotB proteins cycle between these two conformations. Interestingly, the two DotB proteins we have investigated have different hexameric organization, with DotB_L_ consisting of trimer of α–β dimers, whereas DotB_Y_ consists of dimers of α–α–β trimers. These observations suggest a significant degree of conformational variations among the family of VirB11 ATPases.

In previous VirB11 ATPase structures such as HP0525, we have observed ATP‐driven conformational changes that affected subunits in a pairwise fashion, with each subunit cycling through three conformations (apo, ATP‐bound, ADP‐bound) and diametrically opposite subunits adopting the same conformation.^9^ These conformational changes mostly affected the intrasubunit NTD_*n*_/CTD_*n*_ domain orientation and were strictly dependent on ATP‐binding, ‐hydrolysis, and ‐release. Here we observe something altogether completely different: As far as the ATP cycle is concerned, the DotB proteins were captured in the same state (phosphate‐bound for DotB_L_ and apo for DotB_Y_), yet, two widely‐different conformations, α and β, affecting not only the intra‐subunit NTD_*n*_/CTD_*n*_ interface, but also the inter‐subunit NTD_*n*_/CTD_*n*__+1_ or CTD_*n*_/CTD_*n*__+1_ interfaces were observed. The two conformers co‐exist regardless of the nucleotide‐bound states of the proteins. Similar results were obtained in structural investigations of EspE and PilB, emphasizing the close structural similarities of DotB with Type II secretion and Type IV pilus biogenesis ATPases.^12^, ^13^, ^14^ Overall, our DotB structures expand considerably our knowledge of the range of available conformations available to VirB11‐like proteins. It is possible that the transition from α to β might be coupled to the ATP cycle, but it is also plausible that some of these transitions might be driven by association/dissociation with/from other T4sS system components within the larger system. Therefore, our study reveals an unprecedented level of conformational complexity, which might be exploited functionally at many different levels. Such a large conformational spectrum might be the reason why it has been difficult to design VirB11‐targetting drugs.^18^, ^19^, ^20^, ^21^ The novel insights we now provide might prove decisive in progressing these efforts to a successful conclusion.

## Materials and Methods

### DotB_L_ and DotB_Y_ expression and purification

Coding sequences of DotB_L_ and DotB_Y_ were amplified by PCR using *L. pneumophila* DNA (courtesy of Craig Roy) and *Y. pseudotuberculosis* IP31758 plasmid (courtesy of Elisabeth Carniel), and cloned in a pASK vector allowing expression with an N‐terminal StrepTag. Constructs were transformed in *E. coli* C43, and grown in TB medium at 37°C before induction by addition of anhydro‐tetracycline at 18°C. Cells were pelleted by centrifugation, resuspended in a Tris 0.05*M*/NaCl 0.4*M* buffer, and lysed using a C3‐Emulsiflex. The cleared lysates were then applied to a StrepTrap column (GE Healthcare), and the tagged proteins eluted by the addition of des‐thio‐biotin. Concentrated fractions were applied to a gel filtration Superdex 200 16/60 column equilibrated with Tris 0.05*M*, NaCl 0.2*M* and 5% glycerol, where a single peak containing the pure protein was obtained (for the WT protein). Fractions at the peak were pooled and concentrated to 5 mg/mL.

### Crystallization, data collection, and processing

Initial crystallization screens were performed using the sitting‐drop vapor–diffusion technique, by mixing equal volumes (0.2 μL) of protein solution (5 mg/mL) and reservoir at 16°C. For DotB_L_, the protein solution was supplemented with 1 m*M* MgCl_2_ and 1 m*M* AMP–PNP (Sigma Aldrich) just before crystallization and crystals appeared overnight at 16°C against a reservoir solution containing 1.2*M* of Na/K phosphate buffer pH 7.2. For DotB_Y_, crystals appeared overnight at 16°C against a reservoir containing 0.1*M* Na/Cacodylate buffer pH5.5 and 12% PEG 8000. Before data collection, harvested crystals were immersed in a solution containing the precipitant mixture and 10% MDP and cryo‐cooled in liquid nitrogen. All data sets were collected at 100°K. Data on crystals of DotB_L_ and DotB_Y_ were collected at the Diamond I03 beam‐line and I24 beamline, respectively (Diamond Light Source, Didcot, UK). The data sets were indexed, processed and scaled using the XDS package.^22^

### Structure determination and refinement

The DotB_Y_ crystals belonged to the *P*1 space group with a solvent content of 58% for 6 molecules in the asymmetric unit. The structure was determined by molecular replacement using PHASER.^23^ Homology models (obtained using CHAINSAW^24^ from the CCP4 suite^25^) derived from the structures of hp0525, EspE, PilB, PilT, and PilT2 were used as search models. However, none would provide a solution, likely due to the variety of relative positions the N‐ and C‐terminal domains of these proteins can adopt. Therefore, we hypothesized that the N‐ and C‐terminal domains of these proteins might need to be used separately and we used these domain structures in various combinations. *In fine*, the successful search models consisted of the PilT (PDB 2EWV) C‐terminal domain and PilT2 (PDB 5FL3) N‐terminal domain. The coordinates were further improved by cycles of manual rebuilding using COOT^26^ and maximum‐likelihood and TLS refinement using REFMAC^27^ and the PHENIX.^28^ NCS and secondary structure restraints were applied throughout. The final model converged to a final *R*_work_/*R*_free_ of 0.25/0.30 at a resolution of 2.75 Å.

The DotB_L_ crystals belonged to the *P* 1 space group with a solvent content of 47.6% for 12 molecules in the asymmetric unit. The structure was determined by molecular replacement using PHASER and the structure of DotB_Y_ as search model. The coordinates were further improved by cycles of manual rebuilding using COOT^26^ and maximum‐likelihood and TLS refinement using REFMAC^27^ and the PHENIX.^28^ NCS and secondary structure restraints were applied throughout. The final model converged to a final *R*_work_/*R*_free_ of 0.24/0.28 at a resolution of 2.99 Å.

Figures were prepared using PyMol (The PyMOL Molecular Graphics System, Version 2.0 Schrödinger, LLC), and Chimera.^29^

### ATP hydrolysis assays

Proteins were diluted to the desired concentration in the gel filtration buffer supplemented with 2 m*M* MgCl_2_ before the assay. Assays were conducted using the ATPase/GTPase Assay Kit (Sigma Aldrich) according to the manufacturer instructions. Briefly, the enzyme is incubated with freshly prepared ATP (Sigma Aldrich) in a microplate at room temperature before stopping the reaction by the addition of a blocking reagent containing malachite green. This reagent allows the reading of the plate at 600 nm where absorbance is directly proportional to the free phosphate concentration in the mixture. The *K*_m_ and *k*_cat_ were determined using initial speed of ATP hydrolysis measurements with stop point at 0, 0.5, and 5 min, with ATP concentrations of 0.25, 0.5, 1, 2, 4, and 8 m*M* using 5 µ*M* of DotB_L_. Inhibition assays were performed as following: DotB_L_ (2 µM) was preincubated for 10 min with AMP–PNP (0.3, 1, 3, and 10 m*M*) at room temperature before the addition of ATP (4 m*M*). The reaction was stopped by the addition of the malachite green reagent after 20 min. All measurements were done in triplicates.

### Analytic SEC–MALS

Size‐exclusion chromatography (SEC) was performed using a Superdex 200 10/300 Increase column (GE Healthcare), equilibrated with Tris 0.05*M*, NaCl 0.2*M*, and 5% glycerol. Separations were performed at 20°C with a flow rate of 0.6 mL min^−1^ using HPLC (Agilent Technologies 1100 series). The samples (100 µL) were injected at a concentration of 0.4 mg mL^−1^. Online MALS detection was performed with a dawn 8+ detector (Wyatt Technology Corp., Santa Barbara, CA) using a laser emitting at 690 nm and by refractive index measurement using an Optilab T‐rex (Wyatt Technology Corp., Santa Barbara, CA). Data analyses were performed using the Astra software (Wyatt Technology Corp., Santa Barbara, CA).

## Accession Numbers

Coordinates for the DotB_L_ and DotB_Y_ structures have been deposited to the data base (PDB entry codes 6GEB and 6GEF) the ERC Advance grant number 321630 to G. Waksman is referenced here: https://erc.europa.eu/projects-figures/erc-funded-projects/results?search_api_views_fulltext=waksman.

## Supporting information

Supporting Information
