# Supplementary material for: X‐ray crystal structures of the type IVb secretion system DotB ATPases
Source: Protein Sci. 2018 Jul 18;27(8):1464–75. doi: 10.1002/pro.3439 (PMC6153414; doi:10.1002/pro.3439)
Supplement: Supplementary file 1 — Supporting Information [file PRO-27-1464-s001.docx]

**Figure S1: Structural alignments of DotB_L_ subunit with other secretion ATPases.**


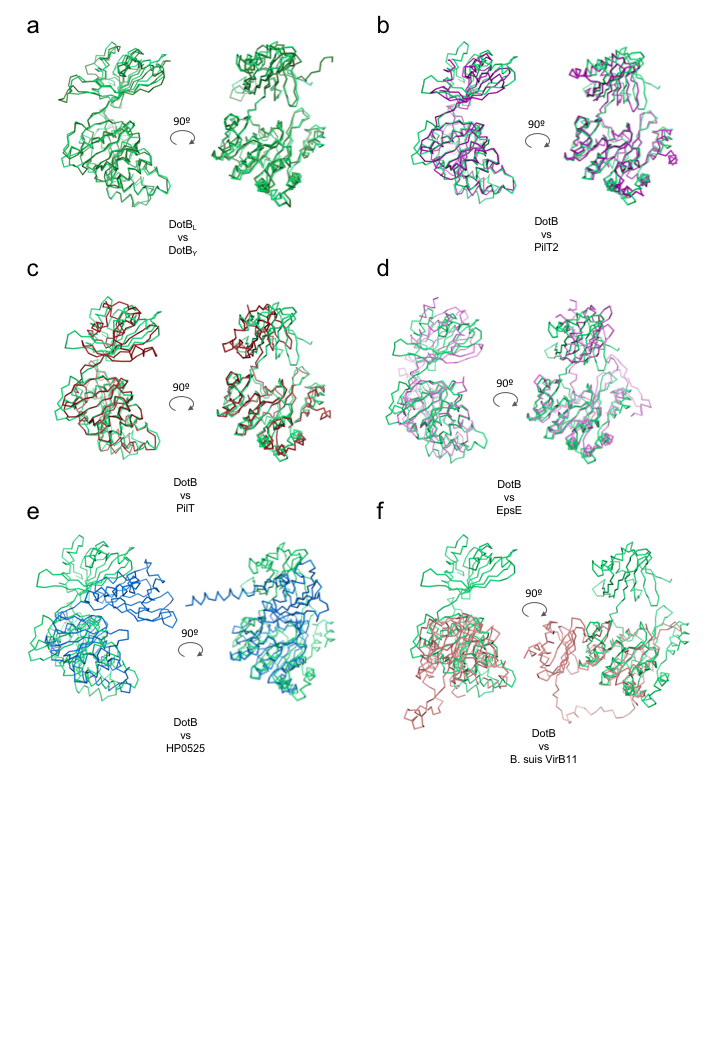


Structures were superimposed using the Cα atoms of the CTDs of the indicated protein. Chain A of DotB was aligned with: DotB_Y_ chain A (a), PilT2 (b, pdb 5FL3), PilT (c, pdb 2EWV), EspE (d, pdb 1PR9), HP0525 (e, pdb 1NLY) and *B. suis* VirB11 (f, pdb 2GZA).

**Figure S2: Sequence alignment of DotB with other secretion ATPases**


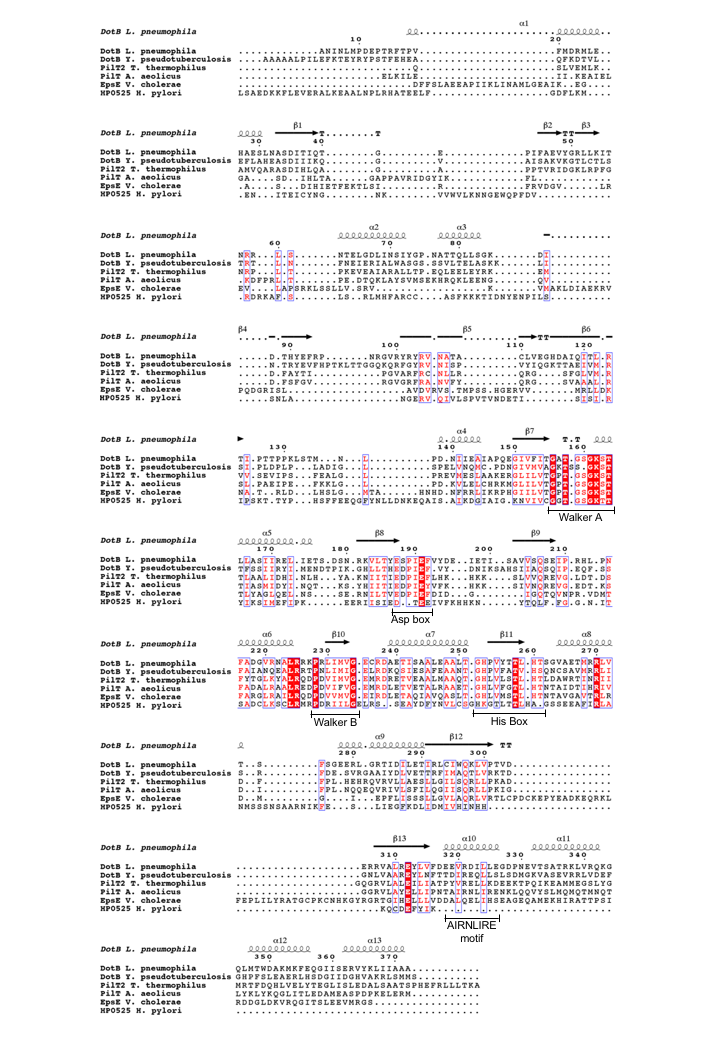


Structure-based sequence alignment of DotB_L_ with DotB_Y_, PilT2 (pdb 5FL3), PilT (pdb 2EWV), EpsE (pdb 1P9R) and HP0525 (pdb 1NLY). This alignment was generated and rendered using DALI ^31^, PDBeFold ^32^ and ESPript ^33^.

**Figure S3: The DotB_Y_ hexamer**

**
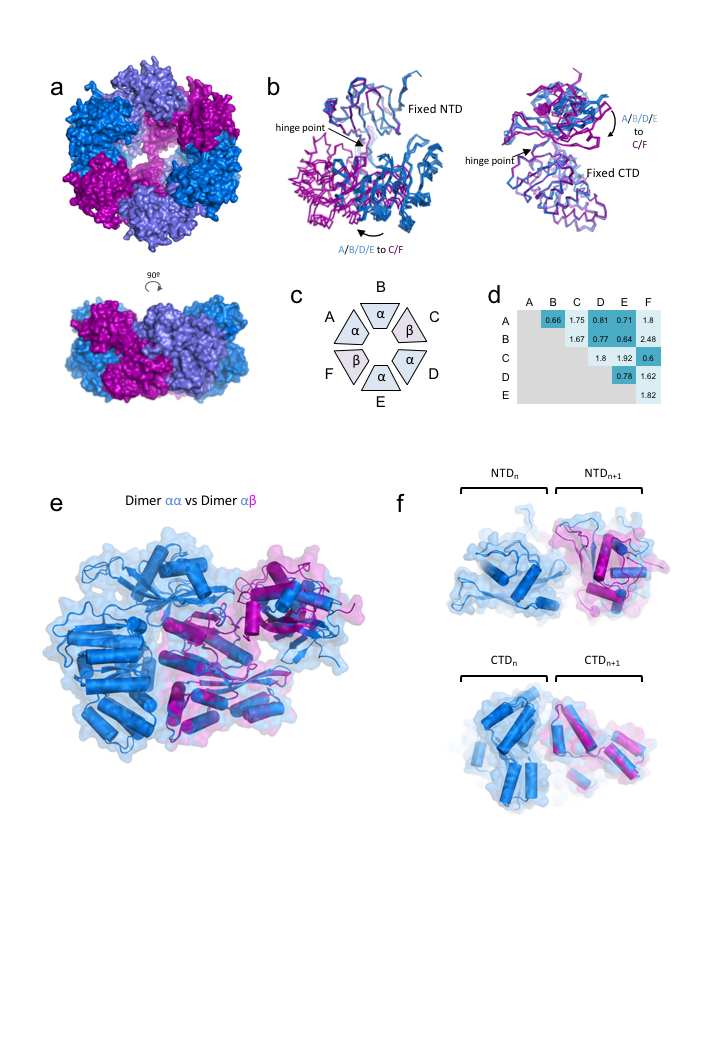
**

**(a)** Top (upper panel) and side (lower panel) views of the DotB_Y_ hexamer in surface representation. Subunits A and D are coloured in marine blue, B and E in grey blue, and C and F in purple.

**(b)** Superposition of the α and β conformers. Subunits are shown in ribbon representation, color-coded in blue and purple for the α and β conformers, respectively. The superposition was obtained by aligning the NTDs (left) or the CTDs (right). The straight and curve arrows identify the hinge point and the direction of the α to β transition, respectively.

**(c)** Schematic representation of the subunits organization within the hexamer.

**(d)** Summary of r.m.s.d. values (Å) resulting from the alignment of the two designated subunits.

**(e)** Superposition of a DotB_Y_ α-α dimer and a DotB_Y_ α-β dimer, using their CTD. The dimers are shown in cartoon and transparent surface representations. α subunits are in blue, the β subunit is in purple.

**(f)** Comparison of the α-α and α-β interfaces, with the same representation as in **e**. Upper panel: the NTD_n_-NTD_n+1_ interface, shown from the top. Lower panel: the CTD_n_-CTD_n+1_ interface, shown from the bottom.

**Figure S4: Competitive inhibition of ATP hydrolysis by AMP-PNP**


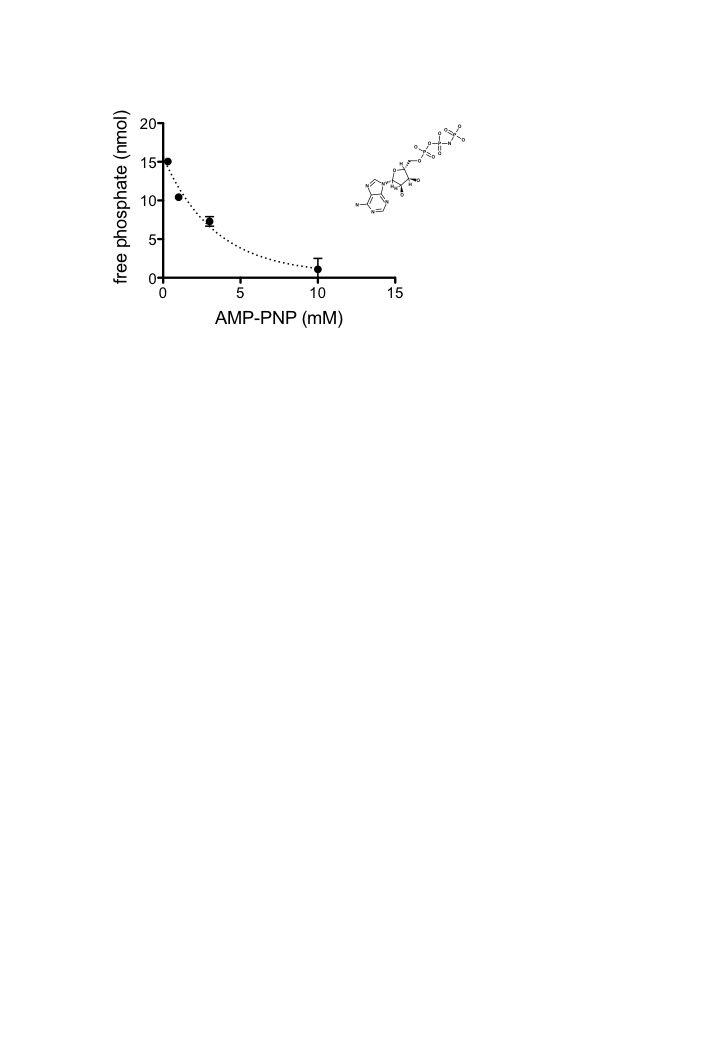


DotB (2µM) was pre-incubated for 10 minutes with AMP-PNP (0.3, 1, 3 and 10 mM) at room temperature before the addition of ATP (4mM). The reaction was stopped by the addition of the malachite green reagent after 20min at room temperature and the free phosphate concentration determined. Error bars are standard deviations of triplicate experiments. The formula of AMP-PNP is shown.

**Movie S1: Model of the α to β conformational transition for the DotB_L_ hexamer**

The movie depicts the result of a Chimera ^29^ morphing between the structure of DotB_L_ and its model where a *n* subunit is in the *n+1* conformation. The hexamer is shown in cartoon representation from the top. Subunits A, C and E are in dark blue while B, D and F are in cyan. The interface between the NTD of chain A and the CTD of chain B is shown is yellow: this region serves a pivot for domain rotation.

**Movie S2: Model of the α to β conformational transition for the DotB_Y_ hexamer**

The movie depicts the result of a Chimera ^29^ morphing between the structure of DotB_Y_ and its model where a *n* subunit is in the *n+1* conformation. The hexamer is shown in cartoon representation from the top. Subunits A and D are in dark blue, B and E in cyan while C and F are in deep green. The interface between the NTD of chain A and the CTD of chain B is shown in yellow.
